# Electrical Resistivity and Carburizing Efficiency of Materials Used in the Cast Iron Melting Process

**DOI:** 10.3390/ma18235413

**Published:** 2025-12-01

**Authors:** Krzysztof Janerka, Jan Jezierski, Mateusz Wojciechowski, Kacper Rosanowski

**Affiliations:** 1 Department of Foundry Engineering, Faculty of Mechanical Engineering, Silesian University of Technology, Towarowa 7, 44-100 Gliwice, Poland; krzysztof.janerka@polsl.pl; 2 Brembo Poland Sp. z o.o., ul. Roździeńskiego 13, 41-308 Dąbrowa Górnicza, Poland

**Keywords:** foundry, carburizing materials, cast iron, carburization efficiency, specific resistivity

## Abstract

The article presents a method of measurement and a test stand for determining the specific electrical resistivity of granular carburizing materials most commonly used in foundry practice. The research was conducted for synthetic graphites (GS) and petroleum cokes (KN) using a test stand proposed by the authors of the study and protected by a patent. It was shown that this measurement method allows for a clear distinction between the tested materials. For synthetic graphites, specific resistivities in the range of 35.9–144.5 μΩ·m were obtained, while for petroleum cokes the range was 172.1–1390 μΩ·m. The main aim of the study was to determine whether there is a correlation between the measured electrical resistivity of the tested materials and the carburization efficiency obtained in melting experiments. Therefore, the article also presents the course and results of studies on the process of cast iron melting in laboratory induction furnaces, where the carburizing material was introduced into the induction furnace with a fixed charge. Carburization efficiencies obtained for synthetic graphite ranged from 86.6% to 94.4%, and from 65.5% to 85.31% for petroleum coke. Based on the measurement results, a statistical analysis was carried out, yielding a relationship with a coefficient of determination R^2^ = 0.92. The research confirmed the possibility of a quick assessment of carburizers in terms of their assimilation degree by molten metal. This is valuable information both for scientific research and industrial applications. The presented results form part of ongoing studies aimed at explaining the differences occurring within a given group of materials (petroleum cokes and synthetic graphites).

## 1. Introduction

Cast iron melting may be carried out in cupola furnaces, induction electric furnaces, or fuel-fired (flame) furnaces. Coke-fired cupolas-occasionally operated on gas-are shaft-type, continuous-operation units with lower energy costs than electric furnaces. They also impart high carbon levels to the iron, since coke functions as both fuel and carburizer. However, cupolas tend to yield iron with elevated sulfur (0.05–0.08%), relatively low superheat, and substantial gaseous and particulate emissions. The high sulfur content confines their use primarily to the production of gray cast iron with flake graphite.

A hybrid (“duplex”) approach-melting in a campaign-type cupola followed by treatment in a medium-frequency induction furnace-can improve product quality. Nevertheless, from a metallurgical-purity and process-control perspective, a standalone induction furnace is optimal. Medium-frequency induction units produce cast iron whose sulfur and phosphorus levels depend solely on charge constituents, allow precise temperature regulation, and, owing to their batch operation, provide the flexibility to cast a wide spectrum of grades, from standard gray irons to high-alloy and ductile irons.

Induction furnaces readily accommodate diverse feedstocks-pig iron, steel scrap, and return cast iron-as well as alloying additions and carburizers. Economic and ecological drivers increasingly favor partial or total replacement of pig iron with scrap metal, aligning with sustainable consumption and production goals. By 2030, these targets promote efficient resource use, waste minimization through prevention, recycling, and reuse, and the deployment of cleaner, more resource-efficient industrial technologies. This scrap-centric strategy also embodies circular-economy principles: in life-cycle assessments, substituting virgin pig iron with recycled scrap markedly reduces the environmental footprint of foundry operations [1].

However, reducing pig iron in the charge necessitates supplementary carbon via external carburizing agents. Synthetic graphite and petroleum coke-often sourced from reclaimed electrode and refractory residues-are the industry’s principal carburizers. These materials typically contain 97–99.5% carbon, with variable sulfur (0.03–0.80%), ash, volatiles, nitrogen, and hydrogen. Although their total carbon contents are similar, their effective carbon transfer to the melt (carburization efficiency) and the kinetics of carbon uptake can differ substantially. Conventional evaluation via preliminary test melts is accurate but costly and time-intensive. Moreover, batch-to-batch variability in carburizer quality can be high, even when sourced from the same supplier.

To address these challenges, the Department of Foundry Engineering at the Silesian University of Technology has developed and patented [2] a rapid screening method based on specific electrical resistivity measurements of loose carburizer powders. In this protocol, lower resistivity correlates with higher carbon-transfer performance. We applied this technique to characterize synthetic graphite and petroleum coke grades intended for metallurgical use. Subsequently, we validated the resistivity–efficiency relationship through gray cast iron melts in a 20 kg induction furnace at the Silesian University of Technology and a 30 kg industrial furnace at Brembo Poland Sp. z o.o. (Dąbrowa Górnicza, Poland). The experimental results and their implications for rapid carburizer screening are presented below.

The authors are not aware of any studies describing the investigation of the properties of carburizing materials using measurements of their electrical resistivity. There are studies presenting other approaches, e.g., [3,4,5,6], but none of them describe solutions similar to those developed by the authors of this publication.

An important stage in explaining the mechanism of graphitization was the work of Rosalind Franklin, who classified carbon materials into two groups—graphitizing and non-graphitizing (susceptible and non-susceptible to the graphitization process).

Characteristic features of non-graphitizing materials include packets of parallel hexagonal layers of small dimensions, spatially disordered, with a large number of cross-links and high porosity (defined as the ratio of pore volume to the total volume of coke including pores, with small pore diameters), which hinders the ordering process.

Examples of such materials include carbon derived from sucrose, polyvinyl chloride coke, charcoal, and bituminous coal with a low degree of carbonization.

In contrast, graphitizing materials exhibit a distinct anisotropic structure, in which numerous layer packets are spatially ordered and interconnected by a small number of weak bonds. This group includes petroleum coke, pitch coke, and coke produced from coking coal [7].

The first theory of the graphitization process, known as the carbide theory, was proposed by Acheson. He assumed that the initial stage of the process involves the formation of metal carbides, which then decompose at high temperatures into gaseous metal and carbon in the form of deposited graphite. To accelerate the formation of graphite, metal oxides such as iron ore and silica were added. Although the decomposition of many metal carbides results in well-crystallized graphite, the carbide decomposition theory is no longer the dominant explanation of the graphitization process.

In practice, the classification of carbon materials as graphitizing or non-graphitizing is difficult. There are cases in which a given carbon material cannot be clearly assigned to either group; therefore, some carbons are classified as transitional [8].

A key parameter in carburization is the rate at which carbon dissolves into the molten metal-known as carburization efficiency [9,10]. This efficiency is governed chiefly by the carburizer’s carbon purity, ash content, and internal microstructure. Of the materials in common use, synthetic graphite offers the highest efficiency, followed by petroleum coke.

Synthetic graphite is manufactured by graphitizing coke or anthracite at high temperatures, during which carbon atoms reorder into the characteristic hexagonal graphite lattice. The process involves three heat-treatment stages-calcination, baking, and graphitization-through which fine-crystalline carbon converts into well-defined graphite. Although numerous studies have elucidated aspects of this transformation, its detailed mechanism remains debated, with prominent models including the Acheson and Franklin theories.

**Figure 1:**
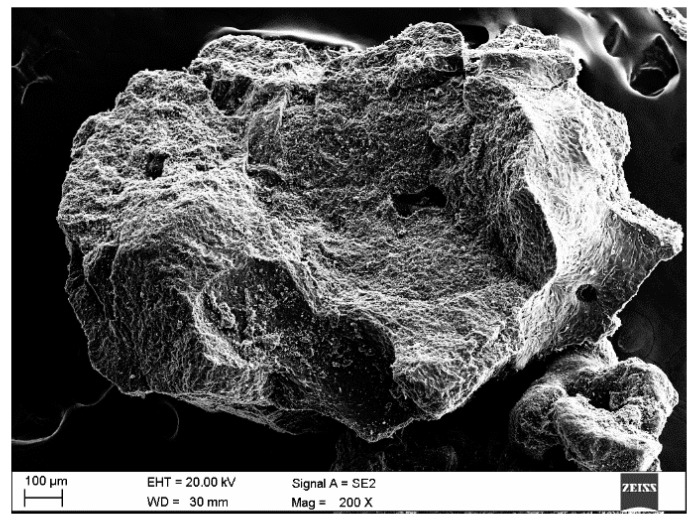

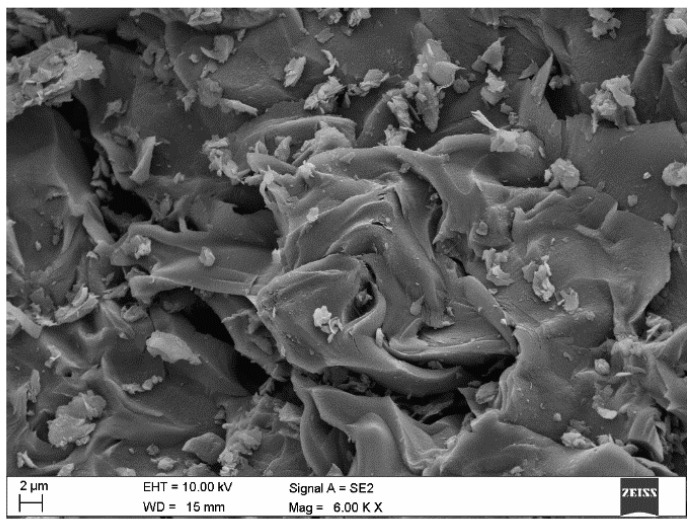
Microstructure of synthetic graphite grains [12].

Typically exceeding 99% carbon with negligible ash, synthetic graphite appears matte and comprises grains of diverse shapes and sizes, with rounded edges and a highly porous surface. Its pores-far larger than those in anthracite or natural graphite-form elongated, ribbon-like channels arranged in either parallel layers or vortex patterns. Figure 1 presents a micrograph of the synthetic graphite used in our experiments. All fracture images were taken using a high-resolution scanning electron microscope SUPRA 35 (ZEISS, Jena, Germany) equipped with an EDS chemical composition analysis system.

Owing to its heterogeneous particle morphology and porosity, this carburizer also exhibits beneficial lubricity. Unlike natural graphite, it lacks a metallic sheen. Its thermal conductivity ranges from 20 to 80 W·m^−1^·K^−1^, and its specific electrical resistivity typically falls between 30 and 200 µΩ·m [11].

Petroleum coke is generated by thermally processing the residual heavy fractions from crude oil distillation. In a typical tubular furnace reactor, the feedstock is rapidly heated to 450–550 °C under 0.1–0.7 MPa, then transferred to an unheated vessel where coke precipitates. The feedstock quality, thermal-cracking conditions, and residence time critically determine the coke’s properties. Sulfur—especially organically bound sulfur—is the most problematic impurity, as it resists removal; accordingly, many grades undergo a subsequent calcination step to volatilize volatiles and reduce sulfur content [3,6].

Chemically, petroleum coke and synthetic graphite are comparable, with carbon contents of approximately 97–99% and 98–99.7%, respectively [9,10,11]. Nevertheless, their carburization efficiencies can differ by up to 20%, owing to structural disparities. Petroleum coke exhibits a highly heterogeneous, irregular microstructure: particles often display wrinkled, rough surfaces and wide variability in size and shape.

**Figure 2:**
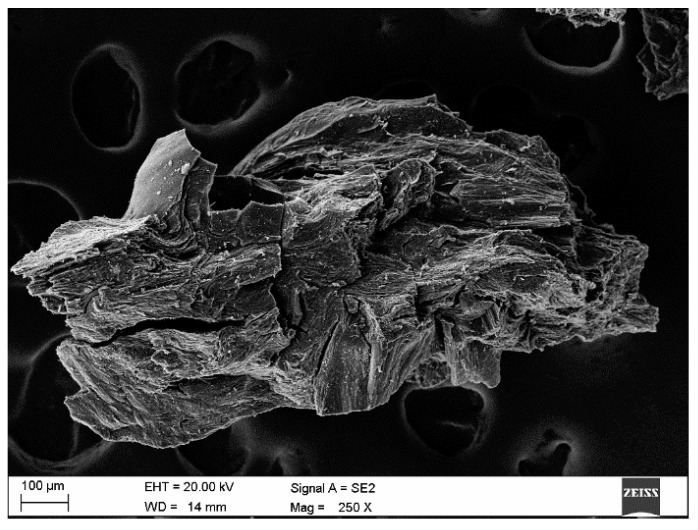

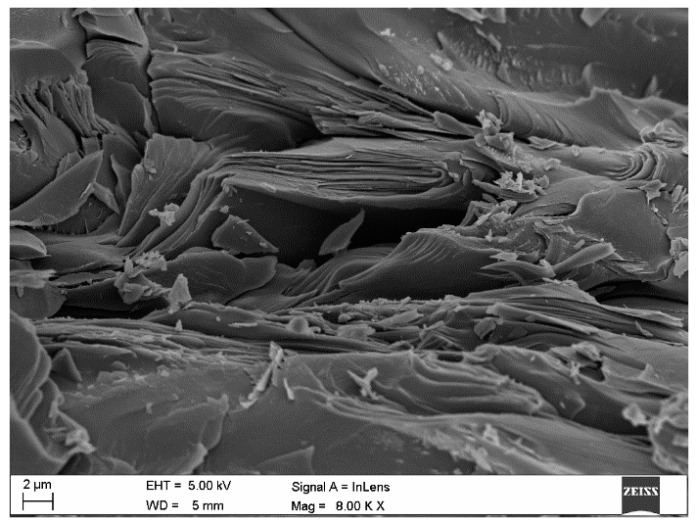
Petroleum coke grains.

Figure 2 shows a representative micrograph of one petroleum–coke sample employed in this investigation.

**Figure 3:**
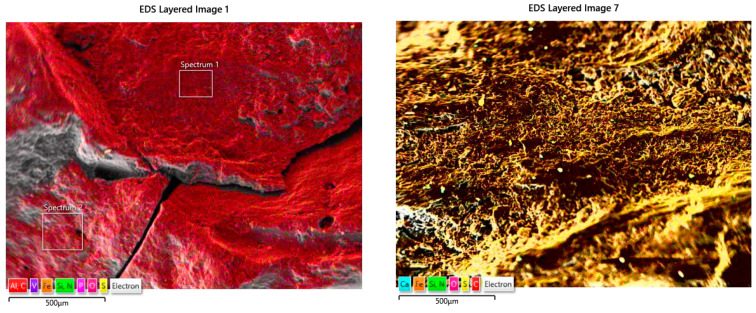
Synthetic graphite (**left**), petroleum coke (**right**). ‘Spectrum 1’ and ‘Spectrum 2’—the areas analyzed for precise chemical compositions—results not included in the article.

The morphology of petroleum coke grains contrasts markedly with that of synthetic graphite: they display far greater heterogeneity, with pronounced irregularities in both shape and surface texture. Further differentiation of these carburizing agents can be achieved via elemental mapping by scanning electron microscopy coupled with energy-dispersive X-ray spectroscopy (SEM-EDS). Figure 3 presents SEM images that compare the surface structures of the petroleum coke and synthetic graphite samples used in this study.

As shown in Figure 3, the surface chemistries of the two carburizers diverge markedly: synthetic graphite grains are nearly pure carbon (red), whereas petroleum coke surfaces contain significant sulfur deposits (yellow).

## 2. Materials and Methods

The authors hypothesize a direct relationship between a carburizer’s carbon-assimilation efficiency in molten iron and its electrical resistivity. Electrical resistivity (specific resistance) quantifies a material’s opposition to electric current; lower resistivity corresponds to easier charge transport and, we propose, more efficient carbon transfer.

Resistivity measurement for consolidated carbon materials (rods, blocks, shaped profiles) is well established [3,6,7,12], and manufacturers routinely specify values. For example, graphite electrodes are classed by power rating: RP (regular power)—8.0–9.0 µΩ·m; HD (high density)—6.0–6.8 µΩ·m; HP (high power)—6.4–6.9 µΩ·m; UHP (ultra-high power)—5.5–6.5 µΩ·m [13].

By contrast, granular carburizers pose unique testing challenges. Although powders can be compacted at pressures up to ~300 MPa into solid specimens, doing so requires specialized high-force presses and binders, which alter the material’s native porosity and resistivity [7,8,12]. Consequently, compacted measurements do not reflect the as-received powder’s behavior.

To overcome this, the authors developed a four-point measurement system that preserves the loose powder’s original structure. A constant-current source (1) drives current through the sample-modeled as an unknown resistor RXR_XRX-in series with a precision reference resistor RrR_rRr (4) and an ammeter (2). The powder is contained within a non-conductive PMMA or polycarbonate tube (12 mm ID) clamped between pneumatic pistons (7, 8) to ensure uniform packing. Three equidistant voltage taps (A1, A2, A3), spaced 30 mm apart along the tube wall (6), accept high-impedance voltmeter probes. By measuring the voltage drop between taps and dividing by the known current, one obtains the specific resistivity of the granular sample directly, without compaction artifacts [11,14].

Pneumatic pistons, sized to the internal diameter of the measurement tube, compress the sample uniformly. The actuators are mounted on a rigid base and fed with compressed air at 1.0 MPa (11). Piston extension and retraction are controlled via a directional control valve (9) connected to the actuators by pneumatic lines (10). Voltage is measured by inserting the voltmeter probes (3) sequentially into the designated ports (6).

**Figure 4:**
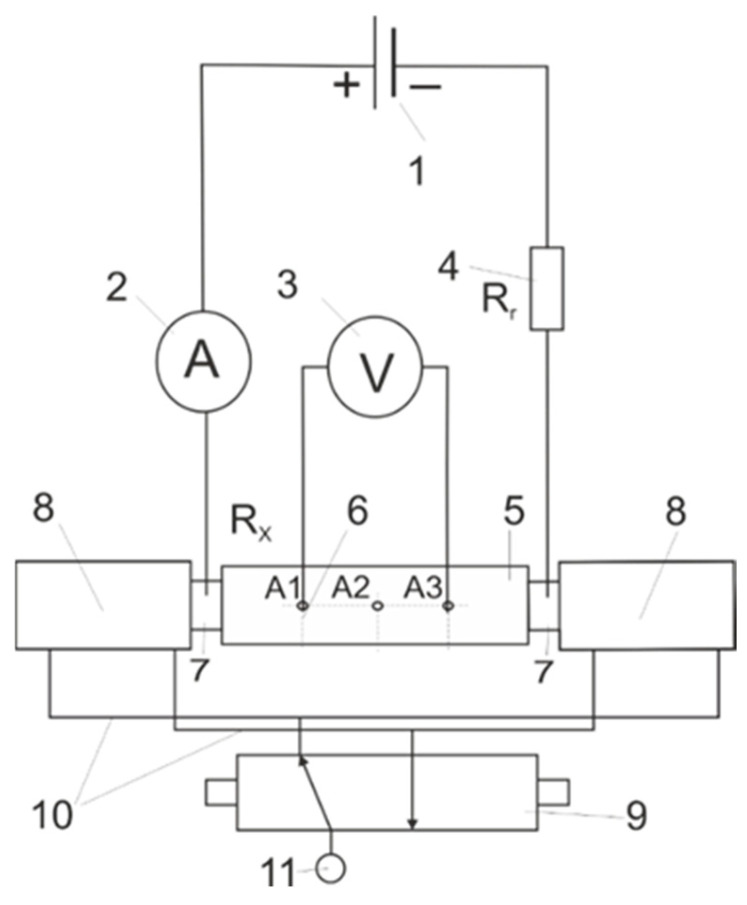

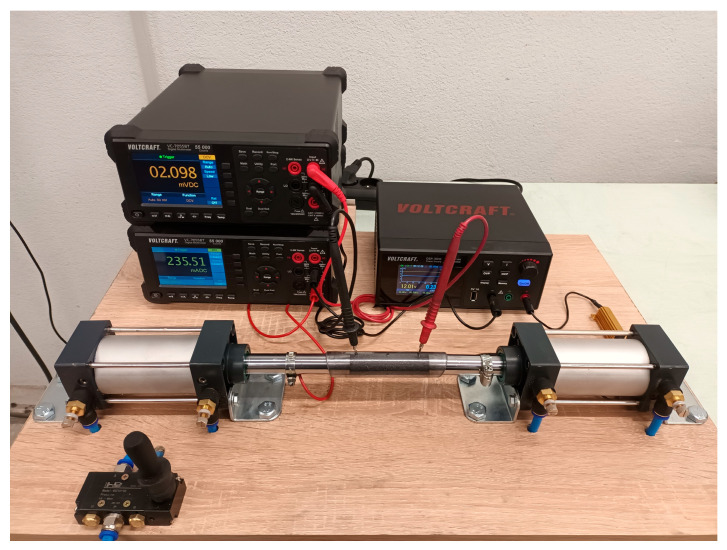
Electrical circuit scheme and assembled device.

A schematic of the apparatus and a photograph of the setup are shown in Figure 4.

**Figure 5:**
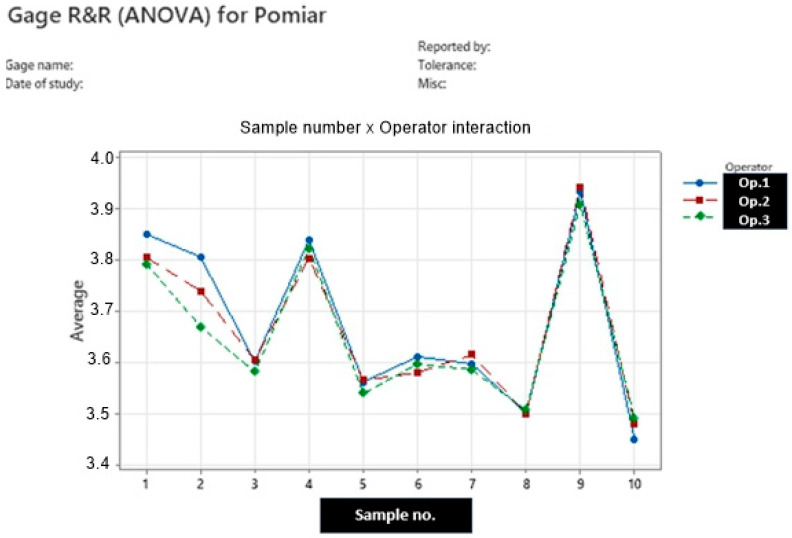
Variability of operators.

Before its use in the ensuing experiment, the measurement system underwent a Measurement System Analysis (MSA) to verify its stability and its fitness for determining the resistivity of carburizing agents. This MSA involved three operators, ten distinct carburizer samples, and three replicate measurements per sample. The resulting analysis of variance (ANOVA) and the operator-variability versus test-part plot are shown in Table 1 and Figure 5.

The results of the repeatability and reproducibility (R&R) analysis of the measurement system indicate that variability attributable to the measurement system and operators accounts for only approximately 5% of the total measurement variation. The dominant source of variation arises from differences among individual samples. Given the negligible influence of both the operator and the measurement system on the results, the setup was considered appropriate for evaluating the carburizing agents in the subsequent experiments.

As part of this study, gray cast iron melting experiments were conducted using a 20 kg laboratory induction furnace at the Department of Foundry Engineering and a 30 kg furnace at Brembo Poland Sp. z o.o. The charge materials included steel scrap, circulating cast iron scrap, FeSi75, and various types of carburizers (as listed in the tables above). The mass of metal used in selected heats (Mm) is presented in the table: steel scrap—100% in heats with an initial carbon content of 0.20–0.21%. In heats where Cp was 2.20%, the charge consisted of 38% steel scrap, 36% cast iron chips, 21% return scrap, and 5% pig iron. In heats 1 and 14–16, 100% low-carbon return scrap was used.

All additives were introduced into the cold charge. The initial carbon content measurement was performed once the molten metal reached a temperature of 1440 °C; this value was then used to calculate the carburization efficiency. Additionally, carbon content measurements were taken at 5, 10, 15, 20, and 25 min after the initial measurement to monitor changes in carbon content during the melt. The carbon content was analyzed using a LECO GDS500A (LECO Corp., St. Joseph, MS, USA) emission spectrometer with a reference database enabling the determination of the chemical composition of monolithic samples of unalloyed and alloyed cast iron, unalloyed and alloyed steel or cast steel, as well as Al and Cu alloys. Additionally, a LECO CS-125 analyzer was used, allowing precise determination of carbon and sulfur concentrations in chips from any metal or alloy.

After melting the metal charge and overheating it to a temperature of 1520 °C, a sample for chemical analysis was taken after 5 min (SAF, manufactured by Heraeus Electro-Nite (Houthalen-Helchteren, Belgium), 35 mm in diameter, 4 mm thick). Samples for spectrometric analysis were ground, while for carbon analysis, chips were taken from solidified samples intended for thermal analysis.

## 3. Results

**Figure 6:**
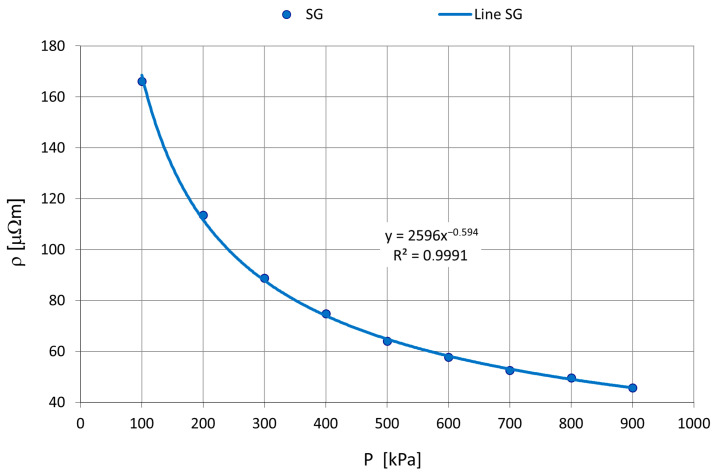
Pressure impact on received electrical values.

Previous studies have demonstrated that the degree of compaction significantly affects the measured values of electrical resistivity [11,15,16]. Therefore, an investigation was carried out to examine the relationship between the applied pressure in the compression cylinders and the specific resistivity of the material. For synthetic graphite, this relationship is illustrated in Figure 6.

For the resistivity measurements, carburizing materials supplied by various vendors were used, including synthetic graphites (SG) and petroleum cokes (PC). A summary of the tested materials, along with their chemical compositions, is presented in Table 2 and Table 3.

### Melting Experiments

**Figure 7:**
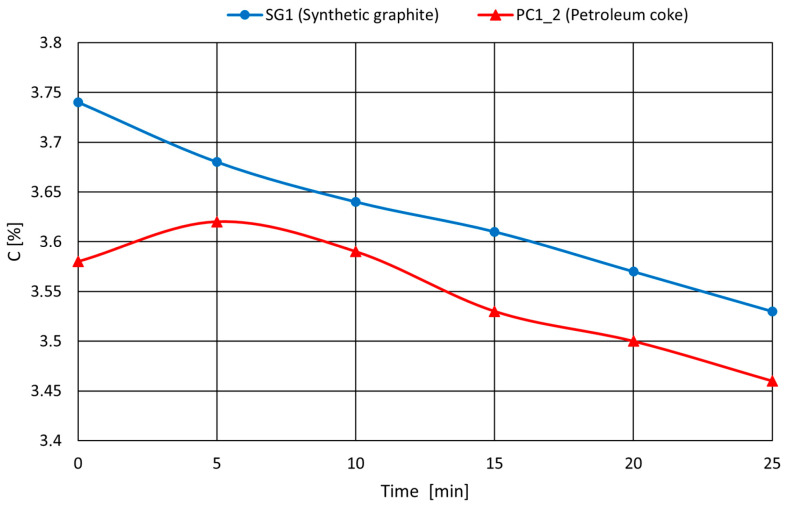
Carbon performance in the melt for selected carburizers; different behaviors observed.

Example results for PC4_2 (petroleum coke) and SG1 (synthetic graphite) are shown in Figure 7.

As shown in the graph above, lower-quality carburizers (such as petroleum cokes) may, in extreme cases, exhibit a tendency for the carbon content to continue increasing during the melting process. This undesirable phenomenon can result in deviations from the intended chemical composition of the final casting, potentially leading to the rejection of castings that fail to meet customer specifications.

In this context, careful quality control of the carburizer prior to its addition to the melt is essential.

Based on the measured electrical resistivity and melting experiments, carbon increments (ΔC) and carburization efficiency (E) were calculated. The efficiency was determined using the following equation:

$$E=\frac{\left(Mm*DC\right)}{\left(Mn*Cmn\right)}$$

where

Mm—mass of the metal,
Mn—mass of the carburizer,
Cmn—carbon content in the carburizer (assumed to be 98% for all materials),
DC—increase in carbon content in cast iron, calculated from the initial carbon content Cp and the final content Ck after melting (Delta C = Ck − Cp). The calculated carburization efficiencies are summarized in Table 4.

**Figure 8:**
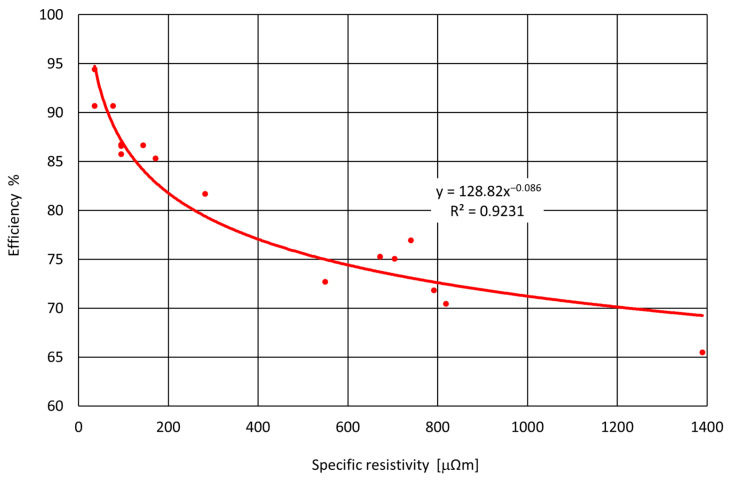
Relationship between performance of carburizers in function of resistivity.

The primary objective of this study was to determine whether a correlation exists between the measured electrical resistivity of the investigated carburizing materials and the carburization efficiency achieved in the melting experiments. The obtained carburization efficiencies and resistivity values are presented in Table 5 and Figure 8, respectively.

## 4. Conclusions

Based on the conducted research, calculations, and obtained results, the following conclusions can be drawn:

1. The investigated carburizing materials (petroleum coke and synthetic graphite) exhibited a wide range of electrical resistivity values, ranging from 36.5 to 1390 mΩ·m.
2. A correlation was established that enables the prediction of carburization efficiency from the measured and calculated electrical resistivity, with a very high coefficient of determination (R^2^).
3. A correlation was established enabling the prediction of carburization efficiency from the measured and calculated electrical resistivity, with a very high coefficient of determination (R^2^).
4. Electrical resistivity measurements are significantly more cost-effective and much less time-consuming (requiring only a few minutes) compared to the costs and time associated with melting experiments and chemical analyses needed to determine the carburization efficiency of production-grade carburizers.

## Figures and Tables

**Table 1:** Variance components.

Source of Variance Comp.	Variance Component	(% of Total Variance)
Total Gage R&R	0.0011996	4.92
Repeatability	0.0006915	2.83
Reproducibility	0.0005081	2.08
Operator	0.0001020	0.42
Operator × sample no.	0.0004062	1.66
Part-To-Part	0.0232028	95.08
Total Variation	0.0244024	100.00

**Table 2:** Carburizers used during the investigation.

No.	Carburizer	C; %	S; %	Ashes; %	VOC; %	Humidity; %
1	SG1	98.5	0.20	0.30	0.60	0.20
2	SG2	99.20	0.05	0.64	0.24	0.30
3	SG3	99.97	0.01	0.03	0.20	0.03
4	SG4_1	99.35	0.04	0.20	0.25	0.10
5	SG4_2	99.35	0.04	0.20	0.25	0.10
6	SG4_3	99.35	0.04	0.20	0.25	0.10
7	SG1_2	98.50	0.28	0.24	0.47	0.01
8	PC1	98.4	1.27	0.2	0.5	0.2
9	PC2	99.25	0.82	0.48	0.27	0.10
10	PC3	98.00	0.6	0.6	1.00	0.50
11	PC4	98.40	1.27	0.22	0.34	0.26
12	PC5	98.20	1.26	1.26	0.25	0.53
13	PC6	98.30	0.86	0.28	0.24	0.47
14	PC7	99.6	0.03	0.3	0.7	0.3
15	PC4_2	98.3	1.26	0.1	0.4	0.3
16	PC1_2	98.3	1.05	0.39	0.35	0.25

**Table 3:** Values received during tests.

No.	Carburizers	Specific Resistivity [μΩ m]
1	SG1	35.92
2	SG2	36.1
3	SG3	76.95
4	SG4_1	95.6
5	SG4_2	95.6
6	SG4_3	95.6
7	SG1_2	144.5
8	PC1	172.1
9	PC2	282.6
10	PC3	550
11	PC4	672.2
12	PC5	704.6
13	PC6	740.6
14	PC7	792.00
15	PC4_2	819.00
16	PC1_2	1390.00

**Table 4:** The calculated carburization efficiencies.

No.	Carburizer	Mm [kg]	Mn [kg]	Cp %	Ck %	E %
1	SG1	29.5	0.165	3.22	3.74	94.39
2	SG2	11.73	0.210	2.20	3.81	90.66
3	SG3	11.46	0.440	0.21	3.69	90.67
4	SG4_1	9.82	0.400	0.21	3.68	85.75
5	SG4_2	10.00	0.400	0.21	3.65	86.56
6	SG4_3	9.60	0.400	0.20	3.79	86.72
7	SG1_2	11.74	0.216	2.20	3.77	86.63
8	PC1	14.12	0.522	0.21	3.34	85.31
9	PC2	9.76	0.400	0.21	3.49	81.67
10	PC3	11.76	0.235	2.20	3.68	75.27
11	PC4	29.5	0.165	3.22	3.62	72.68
12	PC5	11.76	0.241	2.20	3.71	75.03
13	PC6	11.76	0.241	2.20	3.75	76.94
14	PC7	29.5	0.165	3.22	3.62	71.80
15	PC4_2	29.5	0.165	3.22	3.61	70.43
16	PC1_2	29.5	0.165	3.22	3.58	65.48

**Table 5:** Results of performance calculations on specific resistivity.

No.	Carburizer	E %	Specific Resist. [μΩ m]
1	SG1	94.39	35.92
2	SG2	90.66	36.10
3	SG3	90.67	76.95
4	SG4_1	85.75	95.60
5	SG4_2	86.56	95.60
6	SG4_3	86.72	95.60
7	SG1_2	86.63	144.50
8	PC1	85.31	172.10
9	PC2	81.67	282.60
10	PC3	72.68	550.00
11	PC4	75.27	672.20
12	PC5	75.03	704.60
13	PC6	76.94	740.60
14	PC7	71.80	792.00
15	PC4_2	70.43	819.00
16	PC1_2	65.48	1390.00

## Data Availability

The datasets presented in this article are not readily available because the data is part of an ongoing study. Requests to access the datasets should be directed to Department of Foundry Engineering, Faculty of Mechanical Engineering, Silesian University of Technology, Towarowa 7, 44-100 Gliwice, Poland.
